# Lipopolysaccharide Promotes the Proliferation and Differentiation of Goose Embryonic Myoblasts by Promoting Cytokine Expression and Appropriate Apoptosis Processes

**DOI:** 10.3390/vetsci9110615

**Published:** 2022-11-06

**Authors:** Jinhui Wang, Mengsi Fu, Danning Xu, Nan Cao, Wanyan Li, Yunbo Tian, Xumeng Zhang, Yunmao Huang

**Affiliations:** 1 College of Animal Science & Technology, Zhongkai University of Agriculture and Engineering, Guangzhou 510225, China; 2 Guangdong Province Key Laboratory of Waterfowl Healthy Breeding, Guangzhou 510225, China

**Keywords:** lipopolysaccharide, goose, embryonic myoblasts, muscle development

## Abstract

**Simple Summary:**

Lipopolysaccharide (LPS) is the main component of the cell wall of Gram-negative bacteria enriched in polluted water of goose-stocking environments. It can trigger a series of immune reactions, leading to the occurrence of disease and a decrease in the growth performance of geese. This study aimed to investigate the effect and mechanisms of LPS on proliferation and differentiation of goose embryonic myoblasts. As apoptosis processes are strongly linked with myoblasts proliferation and differentiation, compounds that inhibit or promote apoptosis combined with LPS were used to explore the effect of apoptosis on proliferation and differentiation of goose embryonic myoblasts. It was found that LPS promotes the proliferation and differentiation of goose embryonic myoblasts by promoting cytokine expression and appropriate apoptosis processes. These findings lay a foundation for the study of the effects of LPS on goose muscle development.

**Abstract:**

Lipopolysaccharide (LPS) can trigger a series of immune reactions, leading to the occurrence of disease and a decrease in the growth performance of geese. However, the mechanisms of LPS in geese muscle development have not been reported. This study aimed to investigate the effects and mechanisms of LPS on proliferation and differentiation of goose embryonic myoblasts. Embelin and belnacasan combined with LPS were used to explore these effects. Our results demonstrated that LPS significantly induced inflammatory cytokine production in both proliferation and differentiation stages. LPS and embelin treatment significantly improved the proliferation ability (*p* < 0.05), while LPS reduced the differentiation ability of goose embryonic myoblasts. By adding embelin, the differentiation ability of myoblasts was enhanced, while by adding belnacasan, LPS treatment led to a lower differentiation ability. Combined with the correlation of the expression levels of myogenic, cell cycle, and inflammatory-related genes and proteins, it is speculated that one of the reason for the decrease of differentiation ability of goose embryo myoblasts induced by LPS is the increase of the expression levels of pro-inflammatory factors. Moreover, LPS, embelin and belnacasan, and LPS treatments could significantly increase the apoptosis rate of goose embryonic myoblasts. Taken together, these findings suggest that LPS promotes the proliferation and differentiation of goose embryonic myoblasts by promoting cytokine expression and appropriate apoptosis processes. These findings lay a foundation for the study of the mechanisms of LPS in goose muscle development.

## 1. Introduction

Geese are important economic poultry. They have been raised for a long time in China and have a wide distribution range. According to data provided by the China Animal Husbandry Association and the World Food and Agriculture Organization, China is the country with the largest number of meat geese in the world [1]. Meat production is one of the most important factors affecting poultry’s economic benefits, and skeletal muscle development makes a great contribution to meat production. However, the processes of goose stocking are easily affected by environmental pollution, resulting in declined meat production.

Lipopolysaccharide (LPS) is the main component of the cell wall of Gram-negative bacteria. After cell death or artificial lysis, it is released outside of the cell. Its special structure and bioactive parts can be recognized by the immune cells after entering the body [2,3]. It can induce a large number of inflammatory factors, triggering a series of immune reactions and leading to systemic inflammatory diseases [4,5]. LPS is often used as a representative model of inflammation-induced animals [3]. Geese are waterfowls and cannot grow well without water. Most goose farms allow direct emission of goose excrement into the bath water, leading to an increase of the bacterial and LPS content. The geese living in this environment accumulate a large amount of endotoxin in their bodies during the processes of bathing and drinking, resulting in the occurrence of disease and a decrease in growth performance [6].

Current studies on mouse C2C12 cells generally suggest that LPS could induce the mRNA and protein expression of cytokines, such as *TNF-α* [7], *IL-2* [8], *IL-6* [7,9], and *IL-18* [10]. A vast array of literature has established that there is LPS-induced apoptosis, proliferation inhibition, and myotube atrophy in C2C12 cells [11,12,13,14]. However, there are few studies concerning the effects of LPS on growth performance of animals, especially for geese. According to previous reports, the lower the content of LPS in the blood of a gosling, the higher the daily gain and carcass quality. At the same time, the breast and leg muscle rate also increased, while the abdominal fat rate decreased [15]. However, there are few reports on the treatment of goose myoblasts with LPS and the effect of the association between apoptosis, proliferation, and differentiation in the LPS-induced inflammatory environment is also not clear.

Skeletal muscle is one of the most abundant tissue structures in animals and the main source of meat protein in human daily consumption. The development of skeletal muscle in vertebrates includes proliferation of myogenic precursor cells or myoblasts, cell cycle withdrawal, differentiation, fusion, and extension to multinucleated myofibers [16]. The myoblasts differentiate into myotubes and then matured myofibers [17,18]. In addition, cell–cell contact and myoblast apoptosis [19,20,21,22] are necessary conditions for myoblast fusion. The myogenic regulatory factor family (MRFs) (*Myf5, MyoD, Myogenin,* and *MRF4*) is a family of genes that play a decisive role in myogenesis [16]. *Myf5* and *MyoD* are genes that determine the initiation and promote the formation of myoblasts [23,24]. *Myogenin, Myh1,* and *Desmin* play an important role in the differentiation of myoblasts and the maintenance of myofiber homeostasis [16,25]. Intercellular contact and appropriate apoptosis are two necessary conditions for myoblast differentiation [20,22]. Appropriate apoptosis means that moderate cell apoptosis is necessary for myoblast fusion and differentiation, while too much or too little apoptosis would inhibit the abovementioned processes. An increase in the number of cells caused by proliferation is the basis for cell contact and appropriate apoptosis. *IGF-1* is an important positive regulator of myoblast proliferation. *RB1, Cyclin A*, and *Cyclin D1* are important genes that coordinate the cell cycle and have certain inhibitory effects on cell-cycle proliferation [26,27,28].

Our laboratory previously established an isolation and cultivation platform of goose embryonic myoblasts [29]. This study aimed to understand the effect and mechanisms of LPS on proliferation and differentiation of goose embryonic myoblasts, as apoptosis is associated with their proliferation and differentiation. We used two compounds to inhibit or promote apoptosis processes. Embelin was shown to inhibit cell growth and induce apoptosis in cancer cells expressing high levels of XIAP [30,31]. Belnacasan (VX-765) is a caspase-1 inhibitor which could efficiently decrease cell apoptosis [32]. In this study, embelin (50 μM) and belnacasan (40 μM) combined with LPS (100 μg/mL and 500 μg/mL) were used to explore the effect of apoptosis on proliferation and differentiation of goose embryonic myoblasts. This study lays a foundation for the study of the mechanisms of LPS on goose muscle development.

## 2. Materials and Methods

### 2.1. Ethics Statement

This experiment was performed in accordance with the regulations and guidelines of the Animal Care Committee of the Zhongkai University of Agriculture and Engineering, and all efforts were made to minimize animal suffering. All experimental protocols were approved by the Animal Experiment Committee of Zhongkai University of Agriculture and Engineering (NO. 2021112709).

### 2.2. Animals and Embryonic Myoblast Isolation

Magang goose embryos (E15) from Qingyuan Jinyufeng Geese Industry Co., Ltd. were utilized for embryonic myoblast isolation. Firstly, we transferred the embryo to a clean bench, opened the air cell of goose embryo with scissors, took out the goose embryo, transferred it to a culture dish containing 1% double antibiotics (GIBCO, Shanghai, China) phosphate buffer saline (PBS, GIBCO, Shanghai, China), and cleaned and collected the leg muscle. 

We then transferred the collected leg muscle to the new culture dish containing 1% double antibiotics PBS solution, and carefully removed the skeletal muscle and fascia with scissors and forceps. After treatment, the muscle was transferred to a 15 mL centrifuge tube with 1% antibiotic–antimycotic solution (Anti-Anti, Life Technologies, Carlsbad, CA, USA) and PBS solution and washed repeatedly to remove blood cells. Then, we transferred the cleaned muscle tissue, cut into minced meat with clean scissors, to several 1.5 mL centrifugal tubes, and then rolled these with rolling bars. This was followed by digestion with 0.25% trypsin (GIBCO, Shanghai, China) at 37 °C for 5 min, with an increase in digestion time according to the situation. We stopped digestion with Dulbecco’s Modified Eagle Medium: Nutrient Mixture F-12 (DMEM/F-12, GIBCO, Shanghai, China) and a medium of 20% fetal bovine serum (FBS, GIBCO, Auckland, New Zealand) and filtered the material through 70 μM cell sieves. After centrifugation at 4 °C, 100 g for 5 min, the supernatant was discarded. The cells were resuspended with 20% FBS DMEM/F-12 solution and counted with a cell counter. After adjusting the cell concentration to about 1 × 10^6^, the cells were seeded into a cell culture dish for purification. After being cultured at 39 °C, 5% CO_2_ humidified atmosphere for 1 h, the supernatant was transferred to a 12-well cell-culture plate for further culture.

The cells were cultured at 39 °C, 5% CO_2_ humidified atmosphere in proliferation medium (DMEM/F-12 medium containing 20% FBS). When the cell density reached 80–90%, the differentiation medium (DMEM/F-12 medium containing 2% horse serum (HS, GIBCO, LA, CA, USA)) was replaced to induce differentiation.

### 2.3. Cell Treatments

Goose embryonic myoblasts were isolated and purified according to the steps in animal and embryonic myoblast isolation, and then inoculated into 12-well cell culture plate. For the proliferation phase analysis, the cells were precultured in proliferation medium at 39 ℃ and 5% CO_2_ for 24 h. The cells were divided into eight groups: the control group (C1), the low-concentration LPS group (L1 group: wash and replace the proliferation medium with LPS (Serotype O55:B5, Sigma, China) concentration of 100 μg/mL); the high-concentration LPS group (H1 group: wash and replace the proliferation medium with LPS concentration of 500 μg/mL); the embelin-treatment group (EC1 group: wash and replace the proliferation medium with embelin (50 μM)); the belnacasan treatment group (BC1 group: wash and replace the proliferation medium with belnacasan (40 μM)); the belnacasan + low-concentration LPS group (BL1 group: wash and replace the proliferation medium with belnacasan (40 μM) and LPS(100 μg/mL)); the belnacasan + high-concentration LPS group (BH1 group: wash and replace the proliferation medium with belnacasan (40 μM) and LPS (500 μg/mL)).

For differentiation phase analysis, the cells were precultured in proliferation medium at 39 °C and 5% CO_2_ humidified atmosphere for 3 days (cell density was about 80%). The cells were divided into as follows: control group (C2 group: washing and changing the differentiation medium), low-concentration LPS group (L2 group: washing and changing the differentiation medium with LPS concentration of 100 μg/mL), high-concentration LPS group (H2 group: washing and changing the differentiation medium with LPS concentration of 500 μg/mL), embelin-treatment group (EC2 group: wash and replace the differentiation medium with embelin (50 μM)), belnacasan-treatment group (BC2 group: wash and replace the differentiation medium with belnacasan (40 μM)), belnacasan + low-concentration LPS group (BL2 group: wash and replace the differentiation medium with belnacasan (40 μM) and LPS (100 μg/mL)), belnacasan + high-concentration LPS group(BH2 group: wash and replace the differentiation medium with belnacasan (40 μM) and LPS (500 μg/mL)).

Each group of cells was provided with four repeating wells. Cells in each group were cultured at 39 °C and 5% CO_2_ humidified atmosphere. After 0.5 h of culture, cells were collected to detect *TNF-α* mRNA expression level; after 3 h of culture, cells were collected to detect *IL-2*, *IL-6*, and *IL-18* mRNA expression levels; and after 24 h of culture, cells were collected to detect *IGF-1*, *MyoD*, *RB1*, *Cyclin D1* (in the proliferation phase); *Myh1*, *Desmin*, *RB1*, and *Cyclin A* (in the differentiation phase) mRNA expression levels. Protein expression of MyoD (in the proliferation phase) and MyHC (in the differentiation phase) was evaluated by Western blot.

### 2.4. Detection of Myoblast Proliferation Ability

The proliferation cell analysis was performed according to the instructions of the Cell-Light^TM^ EdU Apollo 448 In Vitro kit (Ribobio, Guangzhou, China). After treatment with different concentrations of LPS for 48 h, this was replaced by 50 μm EdU culture medium, cells were kept at 39 °C and 5% CO_2_, humidified atmosphere for 2 h, then fixed and stained with Apollo, and observed by fluorescence microscope (Olympus, Tokyo, Japan, IX73P1F). Photoshop 6.0 was used for image processing. The number of EdU immunofluorescence cells and the total number of cells in the same field of vision were calculated, and the proportion of EdU-stained cells in the total number of cells was calculated.

### 2.5. Detection of Myoblast Differentiation Ability

At the differentiation phase, after cells were processed for 48 h, the myotube fusion rate and myotube diameter was detected by MyHC immunofluorescence. After discarding the cell-culture medium, cells were fixed with 4% paraformaldehyde (Biosharp, Hefei, China) for 10 min and paraformaldehyde was discarded. Cells were washed with PBS, an appropriate amount of Trition X-100 (Beijing Dingguo Changsheng Biotechnology Co., Ltd., Beijing, China) was added for 15 min, the PBS permeating solution was discarded, and 4% bovine albumin (Shanghaiyuanye Bio-Technology Co., Ltd., Songjiang, China) solution added. Cells were sealed at room temperature for 1 h, the sealing solution was then discarded, and an appropriate amount of primary antibody diluent (Sigma, M4276, ISR, 1:400) added. They were then cultured overnight at 4 °C. Next the first antibody was discarded and they were washed with PBS. The second antibody diluent (Abcam, ab150113, USA, 1:1000) was added and they were incubated at room temperature for 1 h. The second antibody was then discarded and they were washed with PBS. Then 1 μL/mL DAPI (Solar bio, Beijing, China) working solution was added and they were incubated at room temperature for 5 min and washed with PBS. Fluorescence quenching: the supernatant was discarded, the anti-fluorescence quenching blocking agent was added, the cell morphology and growth were observed by inverted fluorescence microscope, and the photos were taken. Image J 1.8.0 was used to calculate the proportion of green fluorescence in each image.

### 2.6. Detection of Myoblast Apoptosis Processes

After cells were processed at proliferation or differentiation stages for 36 h, assay of apoptosis detection by Flow cytometry (FACS) was performed. The apoptosis of myoblasts was detected by FITC Annexin V Apoptosis Detection Kit with PI (7Sea Pharma Co., Ltd., Shanghai, China). The cells were digested with 0.25% trypsin, and the digestion was stopped with 20% FBS DMEM/F-12 medium. Cells were centrifuged at 100× *g* for 5 min, and then the cells were collected, the supernatant was discarded, and the cells were washed twice with pre-cooled PBS. The number of cells per tube was adjusted to 0.2–1.0 × 10^6^, and 400 µL 1 × binding buffer was added to resuspend the cells. Next, 5 µL FITC-AnnexinV was added to each tube and reacted at room temperature (25 °C) for 15 min in light. Then, 10 µL PI was added, successively, mixed lightly, and reacted at 4 °C for 5 min without light. After the reaction, the cells could be conducted on the machine for flow cytometry analysis. The appropriate channel was selected (FL1 channel detects FITC, FL3 or FL2 channel detects PI), and, in addition, a blank tube (without fluorescence reagent), a FITC single dye tube (only with FITC reagent), and a PI single dye tube (only with PI reagent) were set for voltage-regulation of flow meter and compensation of the fluorescence channel. The experimental results were analyzed by FlowJO software (Version 7.6.1).

### 2.7. Western Blotting

Proteins were separated using sodium dodecyl sulfate polyacrylamide gel electrophoresis (SDS PAGE) and transferred to polyvinylidene fluoride (PVDF) membranes. After blocking with phosphate-buffered saline with Tween 20 (PBST) containing 5% fat-free milk, PVDF membranes were coincubated with the antibodies: anti-MyoD (1:1000, abcam, ab16148, Cambridge, UK); anti-MyHC (1:10,000, sigma M4276, Burlington, MA, USA); and anti-GAPDH (1:10,000, Abcam, ab181602, UK) at 4 °C overnight. Subsequently, PVDF membranes were incubated with the corresponding secondary antibody at 37 °C for 1 h. Proteins were detected using the ECL kit (Beyotime, Beijing), and visualized using a Tanon-5200 Multi (Tanon, Shanghai, China) device. Densitometry analysis was performed using the Image J software (Version 1.8.0).The original picture of western blots in this study can be found in Supplementary Material (Figure S1).

### 2.8. Quantitative RT-qPCR

The cells were taken out from the cell incubator, the culture medium was sucked out, and the cells were washed twice with PBS buffer. The total RNA was extracted according to the instructions of Trizol (Thermo Fisher, Waltham, MA, USA) reagent. After extraction, the total RNA was dissolved in DEPC water, and the purity and concentration of RNA were measured with full wavelength spectrophotometer. The reverse transcription experiment was carried out in accordance with the reverse transcription kit ReverTra Ace qPCR RT Master Mix with gDNA Remover (TOYOBO, Osaka, Japan) instructions.

By comparing with GenBank in the NCBI database, real-time PCR primers were designed by software Primer Premier (Version 5.0) (Table 1). In this study, GAPDH was used as internal reference gene to detect gene expression by RT-qPCR. The cDNA was used according to the instructions of SYBR Select Master Mix kit (Thermo, USA), 20 μL reaction system was used and prepared on ice. The relative mRNA expression was calculated by the 2^−ΔΔCT^ method.

### 2.9. Statistical Analysis

Three biological replicates were set in each group, and the test data were expressed as “mean ± SEM”. Graphpad Prism software (Version 5.0) was used for statistical analysis of the test data, and the t test was used for difference significance analysis. *p* > 0.05 means that the difference is not significant, *p* < 0.05 means that the difference is significant.

## 3. Results

### 3.1. Exogenous LPS Promoted the Expression of Inflammatory Factors in Goose Embryonic Myoblasts

The mRNA expression of proinflammatory factors was measured by real-time RT-qPCR at different times after LPS treatment: 0.5 h for *TNF-α* and 3 h for *IL-2*, *IL-6* and *IL-18* during the proliferation and differentiation phases. 

**Figure 1:**
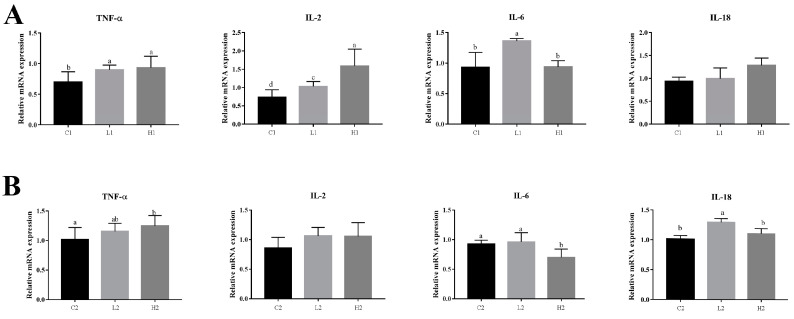
Effect of exogenous LPS treatment on expression of inflammatory factors in goose embryonic myoblasts. (**A**) Relative expression level of *TNF-α*, *IL-2*, *IL-6*, and *IL-18* at the proliferation phase and (**B**) relative expression level of *TNF-α*, *IL-2*, *IL-6*, and *IL-18* at the differentiation phase. Data without common letters represent significant differences (a,b, b,c, or c,d, *p* < 0.05; a–c or a–d, *p* < 0.01). Data are shown as mean ± SEM.

At proliferation phases, the mRNA relative expression of *TNF-α* was significantly higher in groups L1 and H1 than that in group C1 (*p* < 0.05); and the mRNA relative expression of *IL-2* was significantly higher in group H1 than that in groups L1 and C1 (*p* < 0.01), and significantly higher in group L1 than in group C1 (*p* < 0.05); the mRNA relative expression of *IL-6* was significantly higher in group L1 than that in group C1 and H1 (*p* < 0.05); and the mRNA relative expression of *IL-18* showed no significant difference among the three groups (Figure 1A).

At differentiation phases, the mRNA expression level of *TNF-α* was significantly higher in group H2 than that in group C2 (*p* < 0.05); the mRNA expression level of *IL-2* showed no significant difference among the three groups; the mRNA expression level of *IL-6* was significantly higher in group C2 and L2 than that in group H2 (*p* < 0.05); and the mRNA expression level of *IL-18* was significantly higher in group L2 than that in group C2 and H2 (*p* < 0.05) (Figure 1B).

### 3.2. Exogenous LPS Significantly Promoted Proliferation Ability of Goose Embryonic Myoblasts

**Figure 2:**
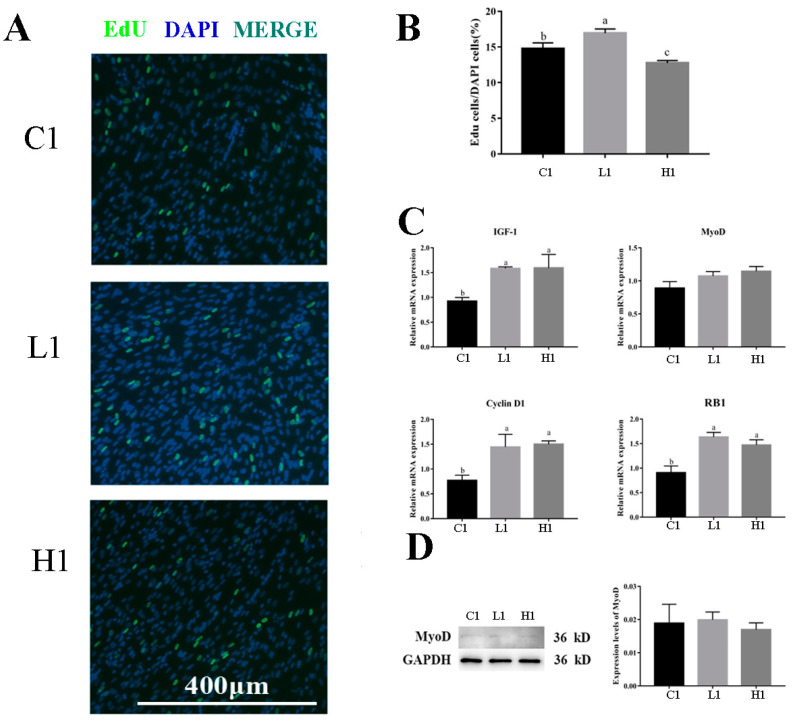
Effect of exogenous LPS on proliferation ability of goose embryonic myoblasts. (**A**) EdU staining results; (**B**) quantitative results of EdU experiment; (**C**) relative expression of *IGF-1, MyoD*, *RB1*, *Cyclin*, and *D1*; and (**D**) Western blot picture and quantitative results of MyoD. Data without common letters represent significant differences (a,b or b,c, *p* < 0.05; a–c, *p* < 0.01). Data are shown as mean ± SEM.

EdU staining revealed that compared with the group C1, the EdU-positive rate of goose embryonic myoblasts in the group L1 was significantly increased (*p* < 0.05) but was significantly reduced in group H1 (*p* < 0.05) (Figure 2A,B).

Myogenic-related gene (*IGF-1*, *MyoD*) and cell cycle-associated gene (*Cyclin D1*, *RB1*) expression was determined by RT-qPCR. The mRNA relative expression of *IGF-1*, *Cyclin D1,* and *RB1* was significantly higher in groups L1 and H1 than those in group C1 (*p* < 0.05); the mRNA relative expression of *MyoD* showed no significant difference among the three groups (Figure 2C).

Western blot analysis was performed to detect the expression of MyoD. The results demonstrate complete concordance of protein expression, as there was no significant difference after LPS treatment (Figure 2D).

### 3.3. Exogenous LPS Significantly Reduced Differentiation Ability of Goose Embryonic Myoblasts

**Figure 3:**
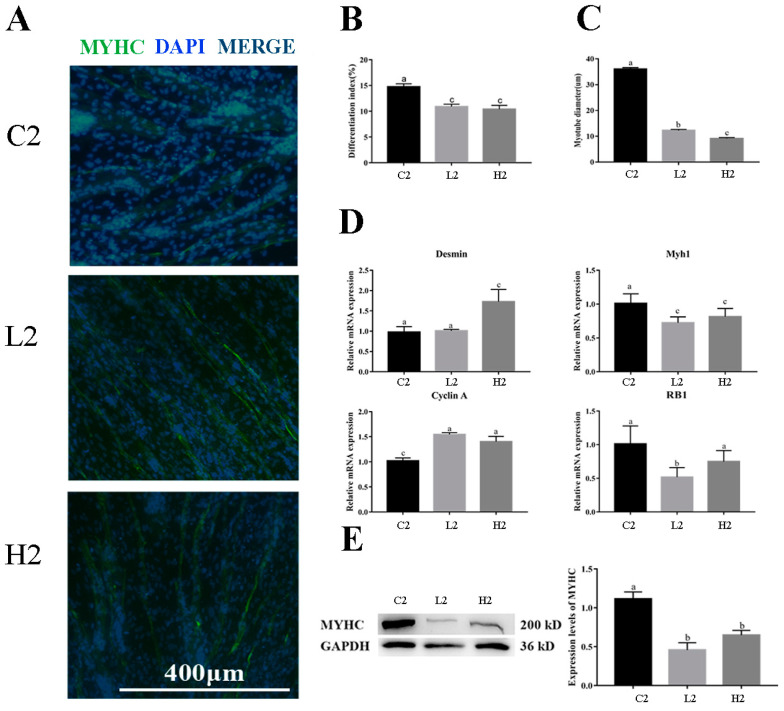
Effect of exogenous LPS on differentiation ability of goose embryonic myoblasts. (**A**) Myogenesis was monitored by MyHC immunofluorescence and DAPI staining; (**B**) quantitative results of myotube fusion rate; (**C**) quantification of myotube diameter; (**D**) relative expression levels of *Myh1*, *Myogenin*, *Desmin*, and *Cyclin A* mRNA; and (**E**) Western blot picture and quantitative results of MyHC. Data without common letters represent significant differences (a,b or b,c, *p* < 0.05; a–c, *p* < 0.01). Data are shown as mean ± SEM.

Immunofluorescence staining of MyHC and myotube diameter was measured to confirm the efficiency of differentiation. The quantification of MyHC immunofluorescence staining results indicated that MyHC protein expression and myotube diameter were significantly decreased after LPS treatment (*p* < 0.01) (Figure 3A–C).

Genes associated with myoblast differentiation (*Desmin*, *Myh1*) and cell-cycle-associated gene (*Cyclin A*, *RB1*) expression was determined by RT-qPCR. The mRNA relative expression of *Desmin* was significantly higher in group H2 than that in groups C2 and L2 (*p* < 0.05); the mRNA relative expression of *Myh1* and *RB1* were significantly higher in group C2 than those in groups L2 and H2 (*p* < 0.05); and the mRNA relative expression of *Cyclin A* was significantly higher in groups L2 and H2 than that in group C2 (*p* < 0.05) (Figure 3D).

Western blot analysis was performed to detect the expression of MyHC. The results show that the protein expression of MyHC was significantly higher in group C2 than that in group L2 and H2 (*p* < 0.05) (Figure 3E).

### 3.4. Exogenous LPS Significantly Promoted Apoptosis Processes in Both Proliferation and Differentiation Phases of Goose Embryonic Myoblasts

**Figure 4:**
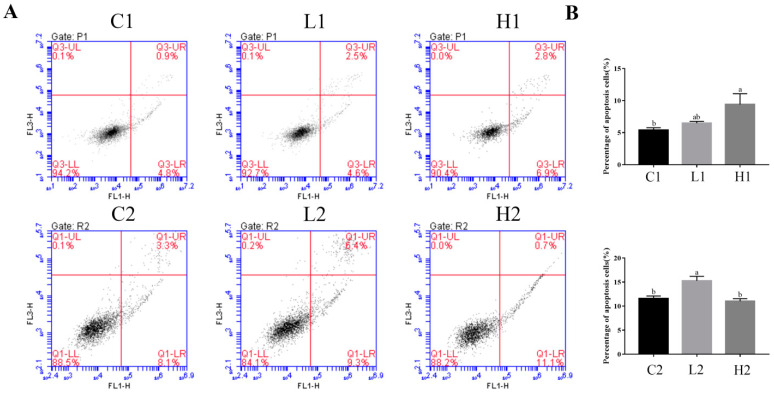
Effect of exogenous LPS on apoptosis processes of goose embryonic myoblasts in both proliferation and differentiation phases. (**A**) Results of FACS in both proliferation and differentiation phases and (**B**) quantitative results of FACS in both proliferation and differentiation phases. Data without common letters represent significant differences (a,b, *p* < 0.05). Data are shown as mean ± SEM.

Flow cytometry with Annexin-V/PI double staining was used to detect apoptosis in goose embryonic myoblasts during the proliferation and differentiation phases. The apoptotic rate of myoblasts was significantly increased in group H1 compared with group C1 (*p* < 0.05) in the proliferation phase. The apoptotic rate of myoblasts was significantly increased in group L2 compared with groups C2 and H2 (*p* < 0.05) in the differentiation phase (Figure 4A,B).

### 3.5. Effect of Embelin or Belnacasan and LPS Treatment on Expression of Inflammatory Cytokines in Goose Embryonic Myoblasts

The mRNA expression of proinflammatory factors was measured by real-time RT-qPCR at different times after LPS treatment: 0.5 h for *TNF-α* and 3 h for *IL-2*, *IL-6*, and *IL-18* during the proliferation and differentiation phases among the five treated groups. 

**Figure 5:**
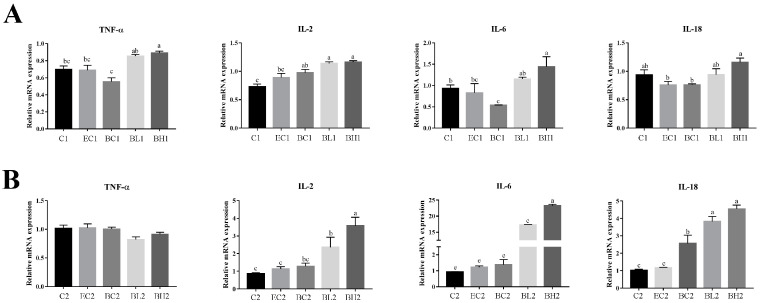
Effect of embelin (50 μM) and belnacasan (40 μM) &and LPS treatment on expression of inflammatory factors in goose embryonic myoblasts. (**A**) Relative expression level of *IL-1β*, *IL-2*, *IL-6*, and *TNF-α* at the proliferation phase and (**B**) relative expression level of *IL-1β*, *IL-2*, *IL-6*, and *TNF-α* at the proliferation phase. Data without common letters represent significant differences (a,b or b,c, *p* < 0.05; a–c, *p* < 0.01). Data are shown as mean ± SEM.

At the proliferation phase, the mRNA expression level of *TNF-α* was significantly higher in group BH1 than it in groups C1, EC1, and BC1 (*p* < 0.05), and was significantly higher in group BL1 than that in group BC1 (*p* < 0.05); the mRNA expression level of *IL-2* was significantly higher in groups BH1 and BL1 than that in groups EC1 and C1, and was significantly higher in group BC1 than that in group C1 (*p* < 0.05); the mRNA expression level of *IL-6* was significantly higher in group BH1 than that in groups EC1, BC1, and C1, and was significantly higher in groups BL1 and C1 than that in group BC1 (*p* < 0.05); and the mRNA expression level of *IL-18* was significantly higher in group BH1 than that in groups EC1 and BC1 (*p* < 0.05) (Figure 5A). 

At the differentiation phase, the mRNA expression level of *TNF-α* showed no significant difference among the five groups; and the mRNA expression level of *IL-2* was significantly higher in group BH2 than that in groups C2, EC2, BC2, and BL2 (*p* < 0.05), and was significantly higher in group BL2 than that in groups C2 and EC2 (*p* < 0.05); the mRNA expression level of *IL-6* was significantly higher in group BH2 than that in groups C2, EC2, BC2, and BL2, and was significantly higher in group BL2 than that in groups C2, EC2, and BC2 (*p* < 0.01); and the mRNA expression level of *IL-18* was significantly higher in groups BH2 and BL2 than that in groups C2, EC2, and BC2, and was significantly higher in group BC2 than that in groups C2 and EC2 (*p* < 0.05) (Figure 5B).

### 3.6. Effect of Embelin or Belnacasan and LPS Treatment on Proliferation Ability of Goose Embryonic Myoblasts

**Figure 6:**
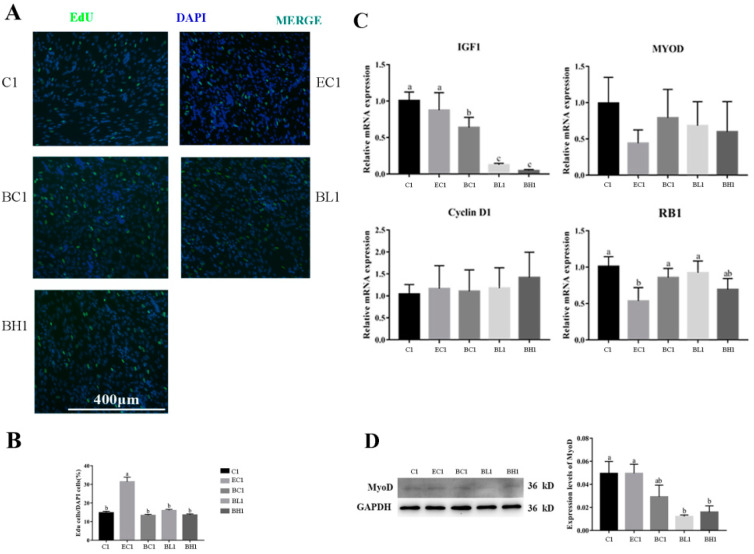
Effect of embelin (50 μM) treatment and belnacasan (40 μM) and LPS treatment on proliferation ability of goose embryonic myoblasts. (**A**) EdU staining results; (**B**) quantitative results of EdU experiment; (**C**) relative expression of *IGF-1*, *MyoD*, *RB1*, and *Cyclin D1*; and (**D**) Western blot picture and quantitative results of MyoD. Data without common letters represent significant differences (a,b or b,c, *p* < 0.05; a–c, *p* < 0.01). Data are shown as mean ± SEM.

EdU staining revealed that the EdU-positive rate of goose embryonic myoblasts in group EC1 was significantly higher in any other group (*p* < 0.05) (Figure 6A,B).

The mRNA expression level of *IGF-1* was significantly higher in groups C1 and EC1 than that in groups BC1, B1L, and BH1, and was significantly higher in group BC1 than that in groups BL1 and BH1 (*p* < 0.05); the mRNA expression level of *RB1* was significantly higher in groups C1, BC1, and BL1 than that in group EC1 (*p* < 0.05); and the mRNA expression of *MyoD* and *Cyclin D1* showed no significant difference among the five groups (Figure 6C).

The results of Western blot analysis shows that the protein expression of MyoD was significantly higher in groups C1 and EC1 than that in groups BL1 and BH1 (*p* < 0.05) (Figure 6D).

### 3.7. Effect of Embelin or Belnacasan and LPS Treatment on Differentiation Ability of Goose Embryonic Myoblasts

**Figure 7:**
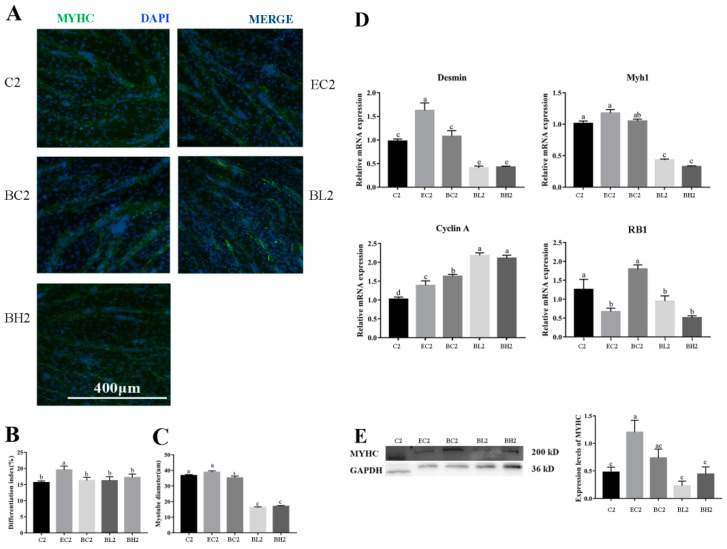
Effects of embelin (50 μM) and belnacasan (40 μM) and LPS treatment on differentiation ability of goose embryonic myoblasts. (**A**) Myogenesis was monitored by MyHC immunofluorescence and DAPI staining; (**B**) quantitative results of myotube fusion rate; (**C**) quantification of myotube diameter; (**D**) relative expression level of *Myh1*, *Myogenin*, and *Desmin* mRNA; and (**E**) Western blot picture and quantitative results of MyHC. Data without common letters represent significant differences (a,b, b,c or c,d, *p* < 0.05; a–c, a–d or b–d, *p* < 0.01). Data are shown as mean ± SEM.

Immunofluorescence staining of MyHC and myotube diameter were measured to confirm the efficiency of differentiation. The quantification of MyHC immunofluorescence-staining results indicated that the immunofluorescence-positive rate was significantly higher in group EC2 than that in other groups, and the myotube diameter was significantly higher in groups C2, EC2, and BC2 than that in groups BL2 and BH2 (*p* < 0.05) (Figure 7A–C).

The mRNA expression level of *Desmin* was significantly higher in group EC2 than that in groups C2, BC2, BL2, and BH2, and significantly higher in groups C2 and BC2 than that in groups BL2 and BH2 (*p* < 0.05); the mRNA expression level of *Myh1* was significantly higher in groups C2, EC2, and BC2 than those in groups BL2 and BH2 (*p* < 0.01); the expression level of *Cyclin A* was significantly higher in groups BL2 and BH2 than in groups C2, EC2, and BC2, group BC2 was significantly higher than groups C2 and EC2, and group EC2 was significantly higher than group C2 (*p* < 0.05); and the mRNA expression level of *RB1* was significantly higher in groups BC2 and C2 than that in groups EC2, BL2, and BH2 (*p* < 0.05) (Figure 7D).

The results of Western blot analysis shows that the protein expression of MyHC was significantly higher in group EC2 than that in groups C2, BL2, and BH2 (*p* < 0.05) (Figure 7D).

### 3.8. Effect of Embelin or Belnacasan and LPS Treatment on Apoptosis Processes of Goose Embryonic Myoblasts

**Figure 8:**
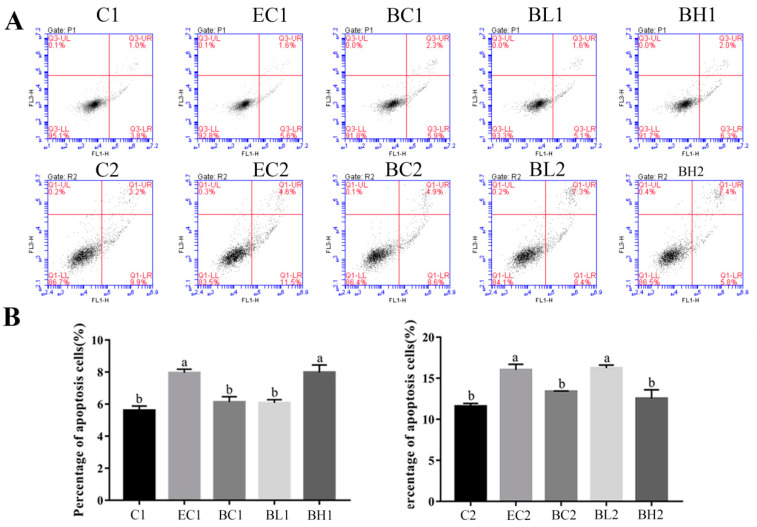
Effect of embelin (50 μM) and belnacasan (40 μM) and LPS treatment on apoptosis processes of goose embryonic myoblasts in both proliferation and differentiation phases. (**A**) Results of FACS in both proliferation and differentiation phases and (**B**) quantitative results of FACS in both proliferation and differentiation phases. Data without common letters represent significant differences (a,b, *p* < 0.05). Data are shown as mean ± SEM.

The results of flow cytometry with Annexin-V/PI double staining showed that the apoptotic rate of myoblasts was significantly increased in groups EC1 and BH1 compared with groups C1, BC1, and BL1 (*p* < 0.05) in the proliferation phase; while the apoptotic rate of myoblasts was significantly increased in groups EC2 and BL2 compared with groups C2, BC2, and BH2 (*p* < 0.05) in the differentiation phase (Figure 8A,B).

## 4. Discussion

In this study, it was found that the addition of exogenous LPS can stimulate increased expression of cytokines in goose embryonic myoblasts (Figure 1), increase their proliferation (Figure 2), and reduced differentiation ability (Figure 3), while promoting the appropriate apoptosis processes (Figure 4). Moreover, embelin or belnacasan and LPS treatment were added to investigate the effects of exogenous LPS-induced apoptosis on the expression of inflammatory factors and the proliferation and differentiation of goose embryonic myoblasts.

There is no doubt that the addition of exogenous LPS can increase the expression of inflammatory factors in goose embryonic myoblasts. Embelin had no significant effect on the expression of inflammatory cytokines in goose embryonic myoblasts. This may be due to the anti-inflammatory effects of embelin [33]. The results showed that belnacasan treatment resulted in changes of inflammatory cytokines in goose embryonic myoblasts and increased the LPS-induced increase in inflammatory cytokines, which was inconsistent with previous results suggesting that belnacasan had anti-inflammatory effects [34,35,36]. 

The results of this study showed that LPS could promote the proliferation of goose embryonic myoblasts while promoting their apoptosis, which was not consistent with previous studies in C2C12 cells [11,37]. However, in other cell studies, LPS promoted the proliferation of the recipient cells by inducing macrophage-derived apoptotic bodies [38]. LPS can also induced proliferation of normal human astrocyte [39] and goat luteinized granulosa cells [40]. Embelin is known to be a potent small molecule inhibitor of the X-linked inhibitor of apoptosis protein (XIAP) that abrogates binding of XIAP to promote caspase-9 expression [41,42], which was often used to induce cell apoptosis [30,31], while belnacasan is a caspase-1 inhibitor which could efficiently decrease cell apoptosis [32]. In our study, embelin promoted apoptosis of goose embryonic myoblasts, while the proliferation rate also increased to a certain extent (Figure 6 and Figure 8). This suggests that appropriate apoptosis can promote goose embryonic myoblast proliferation. The addition of belnacasan had a certain inhibition effect on LPS-induced apoptosis but could not achieve the same effect as the control group. At the same time, LPS-induced increase in myoblast proliferation was not found. It was speculated that the proliferation rate of goose embryonic myoblasts was decreased by the dramatic increase of inflammatory cytokines expression after belnacasan and LPS combined treatment, indicating that appropriate apoptosis of goose embryonic myoblasts can promote proliferation to a certain extent. However, its specific mechanism remains unclear. 

This study found that LPS reduced the differentiation ability of goose embryonic myoblasts. This was consistent with the results of LPS on the C2C12 cell [13,43]. However, in the case of adding embelin, the differentiation ability of myoblasts was enhanced. This may be because embelin not only led to apoptosis of myoblasts, but also increased their proliferation ability. As a result, the number of myoblasts reached a high level, and a high cell density is a necessary condition for myoblast differentiation [20]. In addition, it has been proved that moderate apoptosis also contributes to the differentiation of myoblasts [22]. In our investigation, in the case of adding belnacasan, LPS treatment led to a lower differentiation ability. This may have been due to the adverse effect of higher inflammatory factors on myoblast differentiation [12]. 

## 5. Conclusions

In our study, compounds that inhibit or promote apoptosis combined with LPS were used to explore the effect of apoptosis on proliferation and differentiation of goose embryonic myoblasts. The results indicated that LPS promotes the proliferation and differentiation of goose embryonic myoblasts by promoting cytokine expression and appropriate apoptosis processes. These findings lay a foundation for the study of the mechanisms of LPS on goose muscle development.

## Figures and Tables

**Table 1:** Primer sequences for RT-qPCR.

Gene Name	Forward (5′-3′)	Reverse (3′-5′)
*IL-18*	AAGTGAGGCTCAACATTGCG	CGGTAGAAGATGAAGCGGGT
*IL-2*	ACCGAGAGCTGACCAACTTT	ATCACCCACACTAAGAGCAT
*IL-6*	TTCGACGAGGAGAAATGCTT	CCTTATCGTCGTTGCCAGAT
*TNF-α*	ATGAACCCTCCTCCGTACAC	AGAGGCCACCACATGATAGC
*MyoD*	AAGGCGTGCAAGAGGAAGAC	TGGTTGGGGTTGGTGGA
*IGF-1*	AGGTCGTCCATCGTAGTCCTTGCACTTTTAAGAAGCAATGGA	ACAGCGTCGTTATCGTTCCTGCAAACACAGGCCAAGGTAG
*Rb1*	CCCAGTAGTGAGATTTCTGCT	TGAGCATAGCAGGTGGTGAC
*Cyclin D1*	GCTGCGAAGTGGAAACCATC	CCTCCTTCTGCACACATTTGAA
*Cyclin A*	GGCAGCTCCAACAATCAACC	TGCACAGAGATTCAGGCCAA
*Myh1*	CTCCTCACGCTTTGGTAAAT	GCTCTGGCTTCTTGTTGGAC
*Desmin*	TGAAGGACGAGATGGCCCG	CATCACGGTCTTCTTGGTGTG

## Data Availability

Not applicable.

## Supplementary Materials

The following supporting information can be downloaded at: https://www.mdpi.com/article/10.3390/vetsci9110615/s1. Figure S1: The original picture of western blots.
