# Supplementary figures and images for: Lipopolysaccharide Promotes the Proliferation and Differentiation of Goose Embryonic Myoblasts by Promoting Cytokine Expression and Appropriate Apoptosis Processes
Source: Vet Sci. 2022 Nov 6;9(11):615. doi: 10.3390/vetsci9110615 (PMC9692480; doi:10.3390/vetsci9110615)

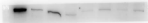

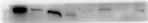

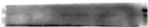

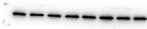

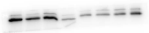

Supplement: Supplementary file 1 [file vetsci-09-00615-s001.zip › Figure S1 The original picture of western.pdf]
